# Seasonal Water-Column Structure Drives the Trophic Niche of Fish Communities on a Temperate Continental Shelf

**DOI:** 10.3390/biology13121041

**Published:** 2024-12-12

**Authors:** Goutam Kumar Kundu, Changseong Kim, Jaebin Jang, Chung Il Lee, Dongyoung Kim, Weol-Ae Lim, Jung Hwa Choi, Chang-Keun Kang

**Affiliations:** 1 School of Earth Sciences and Environmental Engineering, Gwangju Institute of Science and Technology, Gwangju 61005, Republic of Korea; goutam.kundu@du.ac.bd (G.K.K.); changseong@gist.ac.kr (C.K.); wkdwoqls7@gist.ac.kr (J.J.); 2 Department of Fisheries, University of Dhaka, Dhaka 1000, Bangladesh; 3 Department of Marine Bioscience, Gangneung-Wonju National University, Gangneung 25457, Republic of Korea; leeci@gwnu.ac.kr (C.I.L.); dongyoung@gwnu.ac.kr (D.K.); 4 Marine Environment Research Division, National Institute of Fisheries Science, Busan 46083, Republic of Korea; limwa@korea.kr; 5 Ocean and Fisheries Development International Cooperation Institute, Pukyong National University, Busan 48513, Republic of Korea; choi2291@korea.kr

**Keywords:** fish food web, trophic niche, trophic position, benthic–pelagic coupling, Tsushima Warm Current, East China Sea

## Abstract

In seasonally stratified marine environments, the dynamics of benthic–pelagic coupling plays a crucial role in shaping food web structures and fisheries production. We examined fish food web structures across three distinct shelf areas in the Southern Sea of Korea (SSK) during both stratified (summer) and mixed (spring) water conditions using stable isotopes of carbon (δ^13^C) and nitrogen (δ^15^N). In spring, fish communities exhibited a broader range of δ^13^C values compared with summer, indicating more diverse feeding strategies. Seasonal variations in the proportion of benthic and pelagic prey in consumer diets highlighted shifts in benthic–pelagic coupling, illustrating how consumers adjust their reliance on benthic or pelagic resources. The relative importance of the benthic pathway varied among species groups throughout the year. During stratified conditions, reduced benthic–pelagic coupling led to increased reliance on benthic prey, particularly in the oligotrophic region influenced by the Tsushima Warm Current (TWC). The food web spanned five trophic levels, with a median of 3.6. Several species, notably benthic ones, declined in their trophic positions during the summer stratification. These results suggest that fish food webs in the SSK are shaped by temperature-driven seasonal bottom-up control. Our findings further offer insights into how increased water-column stratification could impact the trophic niches of shelf-food webs in the TWC region.

## Simple Summary

Ecosystem processes and energy flow drive marine food web dynamics with primary food sources, trophic interactions, and functional groups are crucial for stability. In continental shelf ecosystems, seasonal benthic–pelagic coupling influences food webs and fisheries, but research on shelf fish responses to environmental shifts is limited. The Southern Sea of Korea (SSK), influenced by the Tsushima Warm Current (TWC) and Changjiang River Diluted Water (CDW), faces rapid warming and seasonal changes that affect productivity and trophic dynamics. Using carbon (δ^13^C) and nitrogen (δ^15^N) stable isotopes, we examined fish trophic structures, revealing distinct seasonal food web patterns. During stratified conditions, reduced benthic–pelagic coupling shifts TWC fish to more pelagic prey, with some species showing lower trophic positions. Benthic fish rely on detrital sources, while pelagic fish are more influenced by pelagic production, especially in spring. Seasonal isotopic shifts reflect changes in pelagic and benthic pathways, with a stronger pelagic influence in spring and reduced resources in summer. Higher productivity in the CDW region leads to more pronounced variations in pelagic consumers, while benthic consumers in TWC regions remain stable. These findings emphasize the need for monitoring warming and TWC influence, which could shift food webs toward greater benthic dependence, impacting fisheries.

## 1. Introduction

Marine food webs are essential for understanding fundamental ecosystem processes, such as the transfer of energy and materials from lower to upper trophic levels and the overall functioning of the ecosystem [1,2,3]. Key properties of food webs, such as primary food sources, the food chain length, interspecific interactions, and functional groups, are essential for assessing how ecosystems respond to environmental changes [4,5,6]. A comprehensive understanding of food web structures and dynamics across spatial and temporal scales is crucial for the sustainable utilization and management of marine ecosystems, especially in the face of environmental changes [7]. Despite extensive global studies of marine ecosystems at various scales [5,8,9,10,11], generalizing ecosystem responses to environmental changes remains challenging because of inherent variability among systems [12]. Consequently, regional studies of marine food webs emphasize differing environmental and organismal structures.

In continental shelf ecosystems, benthic (demersal) food webs integrate photosynthesis-driven pelagic food sources (pelagic pathway) from the surface mixed layer via a vertical flux of organic matter as well as in situ production and detrital recycling (benthic pathway), thereby establishing benthic–pelagic coupling [13,14]. In seasonally stratified temperate seas, the water-column structure significantly plays a crucial role in determining the amount of pelagic production that reaches both benthic–pelagic and benthic food webs [15], as well as nutrient availability to pelagic phytoplankton [16]. Additionally, factors such as feeding strategies, trophic levels, organism body size, and metabolic rates beyond body mass influence the relative contributions of pelagic or benthic resources to specific consumer groups [14,17]. Therefore, the balance between pelagic and benthic pathways affects the overall food web structure and, consequently, fisheries production [18]. Understanding how seasonal variations in water-column conditions influence benthic–pelagic coupling is vital for comprehending marine food web dynamics. However, studies focusing on shelf fish communities and their trophic sources in response to environmental changes are scarce [19].

The East Asian marginal seas, renowned for supporting some of the world’s most productive fisheries [20], are also among the regions experiencing the fastest warming globally [21]. Among these, the Southern Sea of Korea (SSK), forming the northern boundary of the East China Sea (ECS), is particularly important because of its rich biodiversity and significant fisheries [22]. A major characteristic of the SSK is its seasonally dynamic hydrographic conditions [23]. The Tsushima Warm Current (TWC) influences offshore areas during summer and autumn [24], while the Changjiang River Diluted Water (CDW) affects the region following the summer monsoon [25]. A strong summer stratification develops from late spring to autumn (June–November) because of the thermocline formation. In contrast, vertically mixed water masses characterize winter through spring (December–May) because of surface cooling and turbulent mixing [26]. These seasonal changes are associated with shifts in primary productivity and dominant phytoplankton taxa. Additionally, the SSK exhibits a north–south gradient in the bathymetry, sea surface temperature, and nutrient concentrations, which leads to spatial variations in productivity and phytoplankton communities [27,28]. How these spatial and temporal heterogeneities at the base of the food web affect the trophic structures of higher-level consumers, particularly fish, remains largely unexplored.

This study investigates the trophic structures of fish communities across spatial and temporal scales in the SSK, focusing on the role of water-column structures in influencing benthic–pelagic coupling in fish and other nektonic consumers. We expected that seasonal hydrographic conditions in the SSK, including the distribution of distinct water masses (i.e., TWC and CDW) and water-column vertical structures (i.e., seasonal stratification and mixing), would drive changes in primary productivity and dominant phytoplankton taxa composition. Consequently, we further expected that fish communities would exhibit localized adaptations, with variations in trophic niche size, overlap, or specialization across seasons and regions. Stable isotopes (SI) of carbon (δ^13^C) and nitrogen (δ^15^N) were used to trace marine food webs [29,30]. δ^13^C values help distinguish between carbon sources, making it useful for tracing the origins of organic matter and the use of the benthic versus pelagic pathways [13,14]. In contrast, δ^15^N values provide estimates of an organism’s trophic position based on the predictable increases in this isotope from prey to predator [31]. Seasonal and spatial differences at the base of the food chain can cascade to upper trophic levels, providing insights into species-specific and whole food web responses. We hypothesized that changes in environmental conditions between stratified and mixed water-column periods would cause shifts in the isotopic niche of fish communities, as reflected by δ^13^C and δ^15^N values, across three areas of the SSK shelf. To test this, we examined spatial and seasonal differences in δ^13^C and δ^15^N values of organic matter sources, fish, and other nekton. Isotopic niches of fish from pelagic, benthopelagic, and benthic zones were quantitatively compared to illustrate resource utilization patterns. We also estimated the trophic position of individual species to assess trophic structure variations across seasons and regions. Finally, we quantified the contribution of benthic and pelagic food resources to fish production to infer benthic–pelagic coupling and explain seasonal and spatial patterns in trophic structures in the SSK shelf.

## 2. Materials and Methods

### 2.1. Study Area

**Figure 1:**
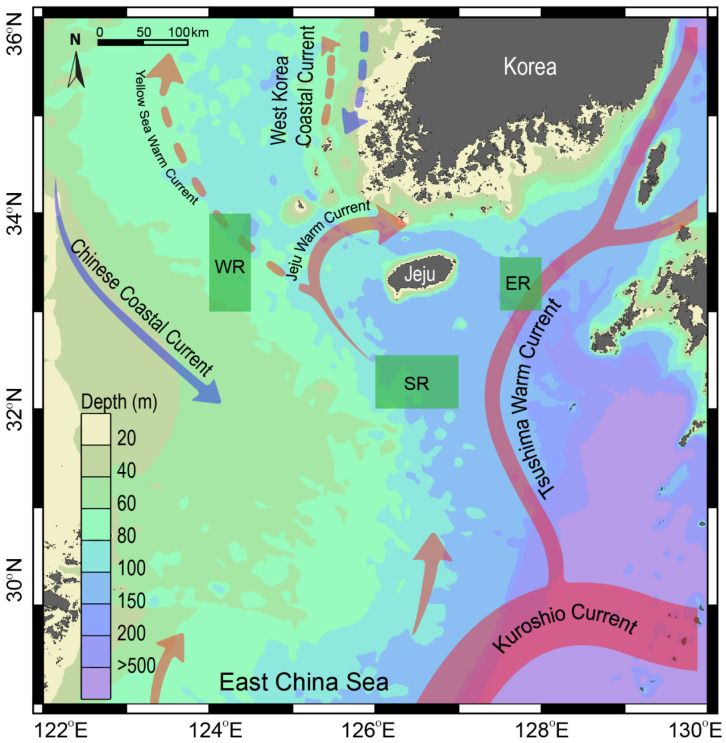
Map showing the study area in the Southern Sea of Korea/northern East China Sea and the three sampling areas to the east (ER), west (WR), and south (SR) off the coast of Jeju Island. Arrows represent the direction of current flow, the solid line color scale indicates the intensity of currents, and the dashed line approximates the water volume of the currents. The map was generated using the Ocean Data view, version 4.7.2 (2015).

This study was conducted on the outer shelf off the coast of Jeju Island in the SSK (Figure 1). The SSK is characterized by a complex hydrographic system with highly seasonal dynamics [23]. As previously described, the study area includes three sampling sites with contrasting water-column structures. The western area (WR) is located on a shallow continental shelf (~60 m depth) and is influenced by the weak Yellow Sea warm current and the Chinese coastal current (Figure 1). This area receives cooler, low-salinity seawater from the CDW, particularly following the summer monsoon from the southwest [25,32]. In contrast, the eastern (ER) and southern areas (SR) are characterized by deeper water masses (100–200 m depth). They are influenced by warm, saline, and oligotrophic waters from the TWC [26]. The influence of the TWC is strongest in summer and gradually diminishes during winter. Chlorophyll *a* concentration in the region ranges from less than 0.4 to approximately 0.4 mg m^−3^ during spring [33] and from 0.2 to 0.3 mg m^−3^ during summer (https://www.meis.go.kr, accessed on 15 November 2024). Primary production, phytoplankton biomass, and particulate organic carbon concentrations decrease progressively from the western basin towards the Kuroshio current region [34]. Given the contrasting environmental conditions across seasons and locations, sampling was conducted in the spring (April–May) and summer (August) of 2018 in the three regions (WR, ER, and SR) off the coast of Jeju Island (Figure 1).

### 2.2. Sample Collection, Processing, and Stable Isotope Analysis

Fish and other nekton specimens were collected from commercial fishing vessels operating within the selected regions. For the isotopic measurement of suspended particulate organic matter (POM) in the water column, water samples were collected 1 m below the surface at each site using a 20 L van Dorn water sampler. Approximately 40 L of water was immediately pre-filtered through a 200 µm Nitex nylon mesh screen to remove zooplankton and large particles. The pre-filtered water was then filtered onto pre-combusted (450 °C for 4 h) Whatman GF/F glass fiber filters (25 mm diameter, 0.7 µm pore size) under a gentle vacuum (150–200 mmHg) on board. POM samples were subsequently frozen on dry ice and transported to the laboratory. Sediment samples for isotopic measurement of sedimentary organic matter (SOM) were collected using a box corer (50 × 50 × 50 cm). The top ~1 cm of the sediment from the box core was scraped off for analysis. Phytoplankton samples were gathered by towing a conical plankton net (20 µm mesh size) until an adequate sample volume was obtained for stable isotope analysis. Zooplankton samples were collected by towing a Bongo net (60 cm mouth diameter, 330 µm mesh) obliquely to a depth of over 40 m. All collected samples were stored on dry ice, transported to the laboratory, and kept in a deep freezer (−20 °C) until further processing.

In the laboratory, microscopic observation separated zooplankton samples into subsamples containing dominant groups (copepods and euphausiids). Fish and other consumer specimens were identified to the species level where possible [35,36] and dissected to collect white muscle tissues. All consumer and source samples were then lyophilized. Dry fish subsamples were defatted using a methanol, chloroform, and water solution (2:1:0.8) [37] to remove isotopically lighter lipid tissues [38]. POM and SOM subsamples were acidified to remove inorganic carbonates: POM by fuming over concentrated HCl for 4 h in a vacuum desiccator, and SOM by adding several drops of 10% HCl solution until bubbling ceased, followed by oven-drying at 50 °C for 48 h. After homogenization by pulverizing, the entire GF/F filter was wrapped in a tin plate, and powdered tissue samples (approximately 1.5 mg) were sealed in tin combustion cups.

The prepared samples were analyzed using an automated elemental analyzer (vario MICRO cube, Hanau, Germany) coupled with a continuous-flow isotope ratio mass spectrometer (IsoPrime 100, Cheadle, UK). Isotope values were expressed in conventional delta notation (δ^13^C and δ^15^N), relative to the Vienna Pee Dee Belemnite for carbon and atmospheric N_2_ for nitrogen, following the equation:

$$\delta X\;\left(‰\right)=(\frac{R_{Sample}}{R_{Standard}}-1)\times 10^{3}$$

where *X* is ^13^C or ^15^N and *R* is the ^13^C/^12^C or ^15^N/^14^N ratio, respectively. International standards (USGS-24 for carbon and IAEA-N1 for nitrogen; NIST, Gaithersburg, MD, USA) were used as reference materials for calibration. Two internal reference material (urea) capsules were analyzed for every 5–10 samples to ensure precision and correctness for potential machine drift. Measurement precision was approximately 0.1‰ for δ^13^C and 0.2‰ for δ^15^N values.

### 2.3. Data Analysis and Statistics

Consumers were classified into three primary feeding zones: pelagic, benthic–pelagic, and benthic. Information on feeding zones was gathered from relevant publications [35] when available; otherwise, data were sourced from FishBase (www.fishbase.org, accessed on 15 November 2022 [39]).

The SI values were normally distributed across stations and seasons (Shapiro–Wilk test, *p* > 0.05) but did not meet the assumption of homogeneity of variances (Levene’s test, *p* < 0.05). Therefore, to assess statistical differences in consumer SI values across spatial and temporal scales, we performed a multivariate analysis using a Euclidean distance-based permutational analysis of variance (PERMANOVA) with the PERMANOVA+ add-on package for PRIMER v6 [40]. Although PERMANOVA does not require normal distribution, it is sensitive to differences in dispersions (specifically, homogeneity of variances). Therefore, homogeneity of dispersions was tested with each PERMANOVA run using PERMDISP [40]. In each analysis, more than 9900 unique permutations were conducted. When significant results were found in the PERMANOVA, they were further explored using a posteriori pairwise comparison with the PERMANOVA *t* statistic [40]. The relative importance of different sources of variation was evaluated based on the square root of the estimates of components of variation. A Euclidean distance-based hierarchical cluster analysis was conducted for each season in a region with the consumer isotope values as the input. Groups were visualized in dendrograms and compared with the feeding zones obtained from previously documented literature.

### 2.4. Trophic Niche Metrics

We quantified and visualized the size, distribution, and overlap of consumer isotopic niches at both the community and feeding-zone group levels using the nicheROVER package [41] (version 1.1.0) in R. This approach incorporates statistical uncertainty through a Bayesian framework, making it robust to variations in sample size and distribution. It also provides asymmetric estimates of niche overlaps, allowing for comparing directional overlaps between pairs of niches. The niche region was defined as the joint probability density function of the δ^13^C and δ^15^N values at a 95% probability level, with ten randomly generated niche regions used for each group. Posterior distributions of niche regions were drawn from 10,000 uninformative random permutations to calculate niche overlap metrics. We report the posterior mean and 95% credible intervals (CI) for all niche overlaps. The estimated niche overlaps were directional, meaning that the overlap of community A with B indicates the probability (%) of finding species from community A within the niche of community B.

### 2.5. Trophic Position and Reliance Measures

The trophic position (TP) of fish and other nekton, as well as their relative reliance on benthic (α) and pelagic (1−α) food sources, were calculated using the R package trophic position [42] (version 0.7.7). Input data included the δ^13^C and δ^15^N values of individual consumer species, one representative primary consumer from each pelagic and benthic habitat as baselines, and the associated trophic enrichment factors (TEF). This approach offers the advantage of providing Bayesian estimates of TP and the contribution of the benthic baseline (baseline 1) to consumers using SIs while accounting for variance in consumers, baselines, and TEF. We used widely accepted TEF values of 3.3 ± 0.7‰ (mean ± 1 SD) for δ^15^N and 1.3 ± 0.30‰ for δ^13^C, based on estimates for muscle tissue isotopes of carnivores [43]. Herbivorous zooplankton (copepods) were used as the pelagic baseline consumer. However, we could not consistently collect deposit-feeding or suspension-feeding benthic consumers across all sampling units for use as benthic baseline consumers. Therefore, we selected invertebrate consumers reported in the literature as exclusive benthic feeders and then chose the consumer with the lowest δ¹⁵N value in each sampling unit (area during each season) as a surrogate for benthic baseline consumers. If the selected benthic baseline consumer was a carnivore or omnivore, we adjusted their stable isotope values by subtracting the aforementioned TEF values prior to model calculations. The baseline consumers and their stable isotope values are detailed in Table S1. We utilized the “twoBaselinesFull” model, which accounts for source heterogeneity by incorporating two baselines (pelagic and benthic) in estimating TP and α. Therefore, 1−α represents the contribution from the pelagic baseline. The model was run with 20,000 iterations and five parallel Markov Chain Monte Carlo simulations, setting the trophic level of baselines (λ) at 2. The estimated median posterior TP and α are presented with a 95% CI. To compare the estimated median TP and α values among areas and feeding-zone groups, we used the Kruskal–Wallis test and a Dunn–Bonferroni post hoc test when significant differences were found. Between-season comparisons were conducted using the Mann–Whitney U Test. In all statistical analyses, a *p*-value of <0.05 was considered significant. Unless otherwise noted, all statistical analyses were performed using IBM SPSS Statistics version 27.0 [44].

## 3. Results

### 3.1. Spatial and Seasonal Patterns in δ^13^C and δ^15^N Values

**Figure 2:**
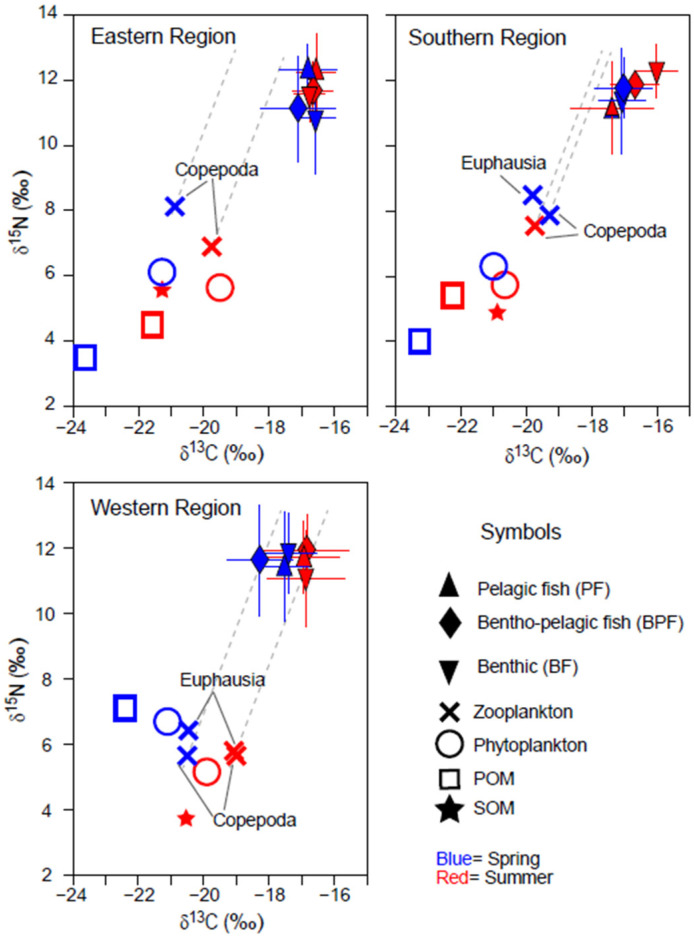
Bi-plots of δ^13^C and δ^15^N values of organic matter sources (phytoplankton, suspended particulate organic matter [POM], and sedimentary organic matter [SOM]) and consumers collected in spring and summer in three areas off the coast of Jeju Island in the Southern Sea of Korea/northern East China Sea. Consumers were categorized based on their primary feeding zone (pelagic, benthopelagic, and benthic). The horizontal and vertical bars represent the standard deviation. The dotted grey lines represent trophic enrichment factors (1.3‰ and 3.3‰ in δ^15^N and δ^13^C per trophic level).

The δ^13^C values of phytoplankton and POM showed narrow ranges of −21.3 to −19.4‰ and −23.7 to −21.5 ‰, respectively, exhibiting similar seasonal and spatial trends (Figure 2). Their δ^13^C values were slightly higher in summer compared with spring in the ER and SR areas, but the seasonal shift was minimal (0.3‰). The δ^15^N values of POM were approximately 2.4‰ lower than those of phytoplankton in ER and SR, while the difference was reduced (<1.3‰) in WR. POM δ^15^N values were slightly lower (~1‰) in spring than in summer in ER and SR but were notably 1.3‰ higher in spring in WR (no data for POM δ^15^N value in WR in Figure 2). SOM δ^13^C values increased by 0.4‰ from ER to WR, while SOM δ^15^N values were significantly lower in WR compared with ER and SR.

The δ^13^C and δ^15^N values of zooplankton followed the spatial and seasonal patterns observed in phytoplankton (Figure 2). Zooplankton δ^15^N values were higher in ER and SR compared with phytoplankton and higher than those in WR. In WR, the δ^15^N values of zooplankton and phytoplankton were nearly identical across both seasons, whereas euphausiids had slightly higher δ^15^N values than copepods.

A total of 95 faunal species, including fish, crustaceans, and cephalopods, were collected across the three areas during both seasons. Of these, 11 species were classified as pelagic feeders, 16 as benthic–pelagic, and 69 as benthic (Table S2). The δ^13^C values of the consumers varied considerably, ranging from −19.4‰ (white-spotted Conger *Conger minister* in the WR during summer) to −14.9‰ (Korean pomfret *Pampus echinogaster* in the WR during summer). Their δ^15^N values ranged from 8.0‰ (Gaper *Champsodon snyderi* in the ER during spring) to 14.9‰ (Japanese seabass *Lateolabrax japonicus* in the WR during spring). Among the three areas, consumers in the WR during spring exhibited the widest range of δ^13^C values (−19.3 to −14.9‰), while those in the ER during summer had a much narrower range (−17.4 to −16.9‰). Cluster analysis based on δ^13^C and δ^15^N values revealed that consumers in each sampling period formed three distinct groups, except in the SR during spring, where four clusters were evident, with benthic fish forming the additional group (Figure S1). Although the isotope-based clustering did not entirely align with previously documented feeding zones (see Materials and Methods, Section 2.3), a noticeable pattern emerged: pelagic and benthic–pelagic consumers tended to group closely together.

A multivariate analysis confirmed significant differences in consumer δ^13^C values across areas (PERMANOVA, Pseudo-*F*_2,217_ = 11.178, *p* < 0.001) and between seasons (Pseudo-*F*_1,217_ = 8.796, *p* = 0.003) (Table 1a). Pairwise comparisons revealed a notable distinction in δ^13^C values between the ER and WR regions (Table 1b). Although the interaction effects among region, season, and feeding-zone group combinations were not statistically significant (PERMANOVA, Pseudo-*F*_2,217_ = 1.548, *p* = 0.192), a more pronounced seasonal difference was observed in the WR (Figure 2). There were also significant differences in δ^13^C values among feeding-zone groups (pelagic, benthic–pelagic, and benthic feeders) (Pseudo-*F*_2,217_ = 5.748, *p* = 0.015), with pelagic fish showing significantly different δ^13^C values compared with benthic groups (Table 1b). In contrast, δ^15^N values of consumers showed no significant variation across areas (PERMANOVA, Pseudo-*F*_2,217_ = 0.0495, *p* = 0.613), seasons (Pseudo-*F*_1,217_ = 2.457, *p* = 0.117), or feeding-zone groups (Pseudo-*F*_2,217_ = 0.702, *p* = 0.504) (Table 1c).

### 3.2. Spatial and Seasonal Pattern in Isotopic Niche of Fish

**Figure 3:**
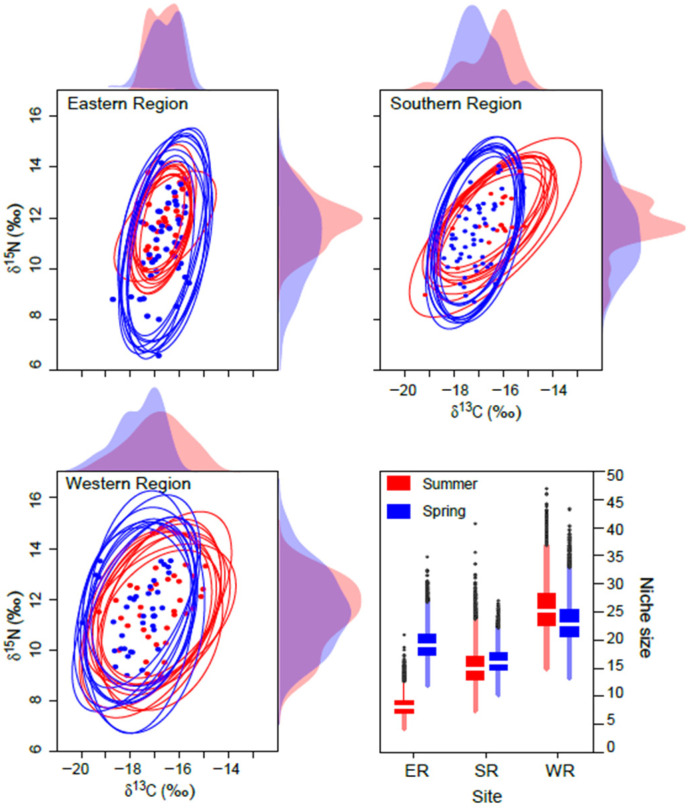
Isotopic niche of consumers in the Southern Sea of Korea in spring and summer. Niche regions were obtained from ten random two-dimensional elliptical projections covering 95% probabilistic region based on δ^13^C and δ^15^N values. Dotted symbols inside the ellipses represent the raw δ^13^C and δ^15^N values. The plots along the axis are one-dimensional density distributions of δ^13^C and δ^15^N values. The box plot represents the estimates of isotopic niche region size (mean ± 1 SD).

**Figure 4:**
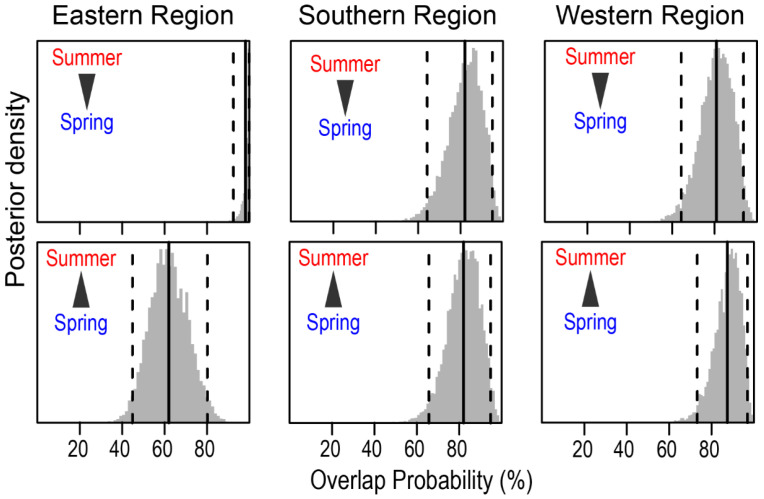
Posterior distribution of the probabilistic niche overlap (%) between consumer’s niche (N_R_ of 95%) of two seasons from the Southern Sea of Korea. The vertical solid lines represent posterior means and dotted lines represent 95% CI. The arrows indicate the directions of niche overlaps between the communities.

The mean probabilistic niche regions (95% level of inclusion) of faunal communities ranged from 8.2 ± 2.8 (narrow niche: ER in summer) to 25.7 ± 4.4 (broad niche: WR in summer) (Figure 3). The total isotopic niche size increased progressively from ER to WR in summer. However, this pattern shifted in spring, with the niche size in the ER community approximately doubling compared with summer, primarily because of changes in the distribution along the δ^15^N axis. In contrast, the isotopic niche sizes in WR and SR remained consistent between seasons. The isotopic niches of faunal communities exhibited substantial overlap between seasons. The estimates of niche overlap showed that the extent of seasonal niche overlap varied by area (Figure 4). In SR and WR, the isotopic niches overlapped extensively between seasons (mean probability > 80%, CI: 65.0–96.8%), while in ER, the overlap was considerable (62.4%, CI: 46.1–80.0%) but notably reduced.

**Figure 5:**
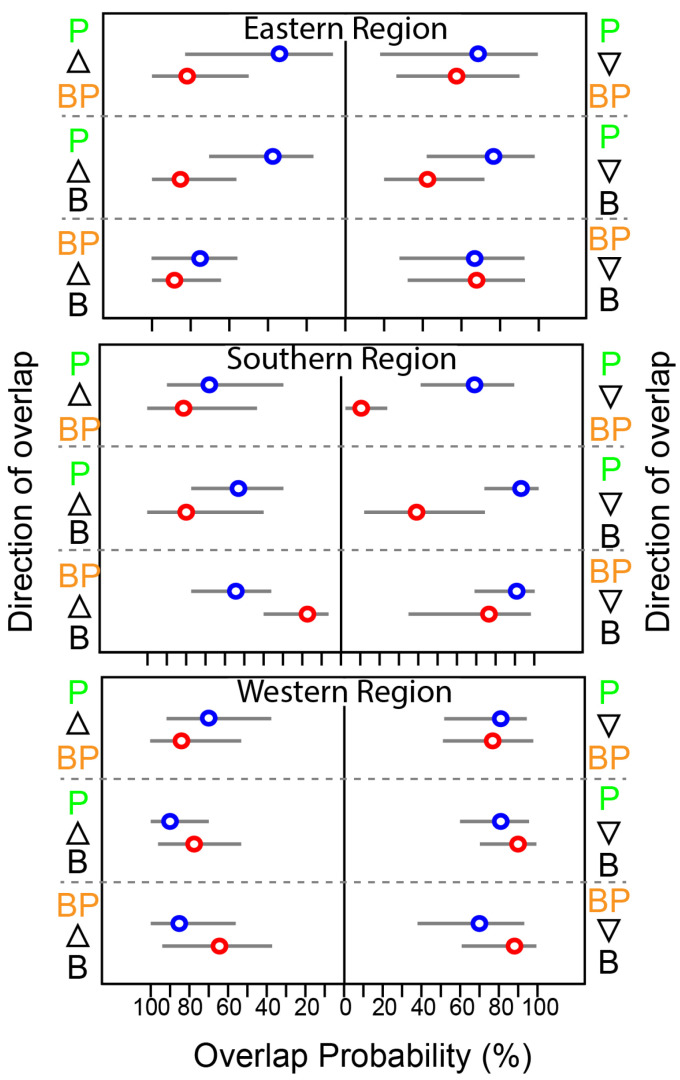
Seasonal variation in the probabilistic niche overlap (%) among the isotopic niche (N_R_ of 95%) of three groups of consumers from the Southern Sea of Korea. The circles represent the posterior mean overlap (%), and the horizontal lines represent the 95% CI. P, B, and BP stand for pelagic, benthic, and benthopelagic consumers, respectively.

At the feeding-zone group level, the extent of niche overlap varied significantly within each community and showed pronounced seasonal differences (Figure 5 and Figure S1). In WR, the overlap between feeding-zone groups exhibited minor seasonal variation (<10%). However, there was substantial seasonal variation in the niche overlap involving pelagic fish and the other two groups in SR. During summer, the probability of benthopelagic and benthic consumers overlapping with pelagic consumers increased substantially (~50%). In contrast, the probability of pelagic fish overlapping with benthopelagic and benthic consumers decreased considerably, particularly in SR (Figure 5). Benthic and benthic–pelagic consumers consistently showed high overlap across seasons and areas.

### 3.3. Trophic Position of Fish

The median TP of all communities was 3.6, ranging from 2.8 to 4.5, indicating a food web spanning five trophic levels, including primary consumers (Table S2). The lowest TP values in each community were mostly associated with benthic invertebrates, such as the Japanese snapping shrimp (*Alpheus japonicas,* TP = 3.0), the Gold-spot octopus (*Amphioctopus fangsiao*, TP = 2.8), and the Shiba shrimp (*Metapenaeus joyneri*, TP = 3.1). Among pelagic consumers, the Japanese anchovy (*Engraulis japonicas*, TP = 3.0) and the Chub mackerel (*Scomber japonicas*, TP = 2.9) had relatively low TPs. The highest TPs were observed in benthic and benthopelagic predatory fish, such as *Pagrus major* (TP = 4.4), *Lateolabrax japonicus* (TP = 4.5), and *Miichthys miiuy* (TP = 4.4). Most benthopelagic fish exhibited relatively high TPs, except for the Pacific rudderfish (*Glossanodon semifasciatus*, TP = 2.8). However, there was no statistically significant difference in TP among the feeding-zone groups (Kruskal–Wallis test, *p* = 0.896). Additionally, TP distributions were not significantly different among feeding-zone groups (Kruskal–Wallis test, *p* = 0.132) or between seasons (Mann–Whitney test, *p* = 0.705).

**Figure 6:**
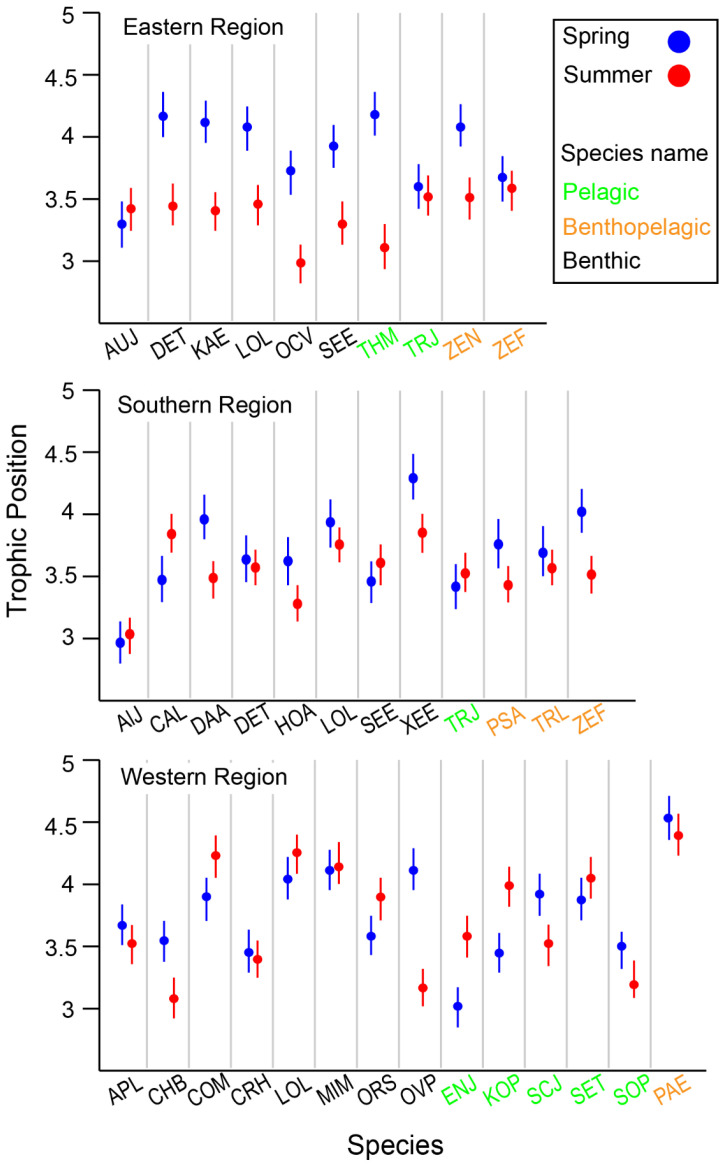
Variation in estimated trophic position (TP) of selected species collected from the Southern Sea of Korea. Species codes, Eastern Region: AUJ *Aulopus japonicas*, DET *Dentex tumifrons*, KAE *Kaiwarinus eqquula*, LOL *Lophius litulon*, OCV *Octopus vulgaris*, SEE *Sepia esculenta*, THM *Thunnus hynnus*, TRJ *Trachurus japonicas*, ZEN *Zenopsis nebulosi*, ZEF *Zeus faber*; Souther Region: AlJ *Alpheus japonicas*, CAL *Carcinoplax longimana*, DAA *Dardanus arrosa*, DET *Dentex tumifrons*, HOA *Hoplobrotula armata*, LOL *Lophius litulon*, SEE *Sepia esculenta*, XEE *Xenocephalus elongates*, TRJ *Trachurus japonicas*, PSA *Psenopsis anomala*, TRL *Trichiurus lepturus*, ZEF *Zeus faber*; Western Region: APL *Apogon lineatus*, CHB *Charybdis bimaculata*, COM *Conger myriaster*, CRH *Crangon hakodatei*, LOL *Lophius litulon*, MIM *Miichthys miiuy*, ORS *Oratosquilla* sp., OVP *Ovalipes punctatus*, ENJ *Engraulis japonicas*, KOP *Konosirus punctatus*, SCJ *Scomber japonicas*, SET *Setipinna tenuifilis*, SOP *Solenocera prominentis*, PAE *Pampus echinogaster*.

When comparing species collected in both seasons within each area, species-specific seasonal variation in TPs was observed, with the variation differing across locations (Figure 6). Overall, benthic consumers in the deeper ER and SR areas had lower TPs in summer compared with spring. In contrast, many benthic consumers exhibited lower TPs in the shallower WR in spring. Benthic–pelagic fish generally showed an increase in TP during spring across all areas, although this observation was not fully conclusive due to limited data availability (fewer than three cases). Among pelagic consumers, seasonal changes in TP were highly variable between species, with no clear patterns emerging.

### 3.4. Contribution of Benthic Pathway to Fish Nutrition

**Figure 7:**
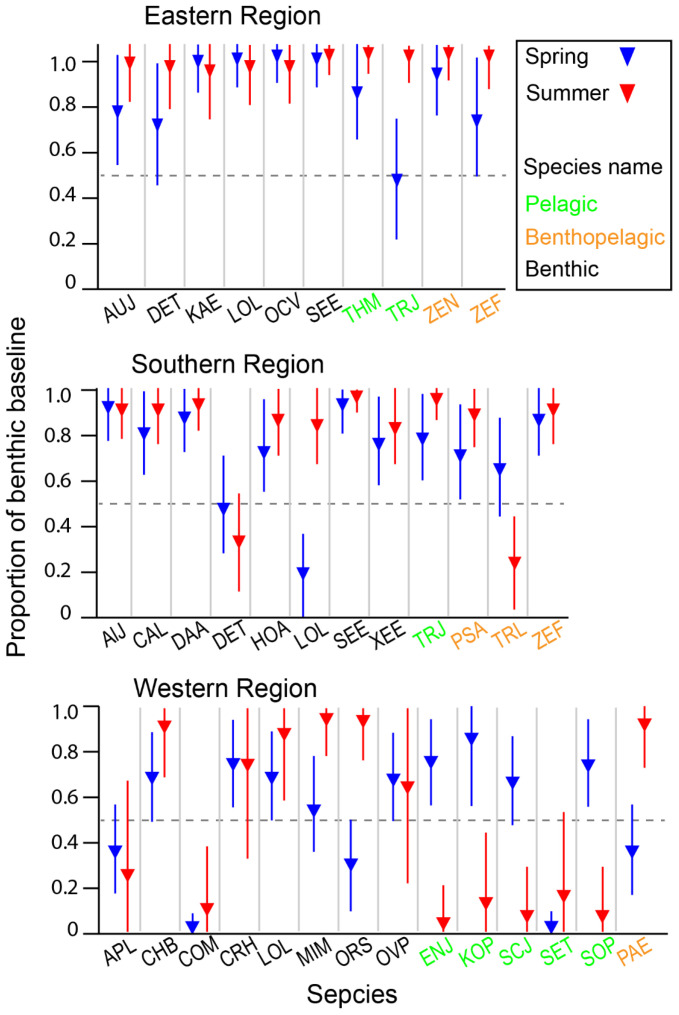
The estimated relative contribution of the benthic pathway to consumer nutrition based on a two-source Bayesian mixing model using δ^13^C and δ^15^N values. Points show the estimated mean, and the vertical bars represent the 95% credible intervals of the posterior distribution. See Table S3 for the posterior distribution of individual species. Species cods are in Figure 6.

The estimated α values for consumers spanned the full possible range from 0 to 1, with a global median estimate of 0.8, indicating that most species predominantly relied on the benthic pathway for nutrition (Table S3). The distribution of α values varied significantly among areas and feeding-zone groups (Kruskal–Wallis test, *p* = 0.000 and *p* = 0.014 for the area and feeding-zone groups, respectively). Among the areas, α values increased in the order of WR, SR, and ER (Dunn–Bonferroni pairwise comparison, *p* < 0.050 for all cases). Regarding feeding-zone groups, benthic consumers showed significantly higher α values than pelagic consumers (Dunn–Bonferroni pairwise comparison, *p* = 0.020). In contrast, the reliance of benthic–pelagic consumers on the benthic pathway did not significantly differ from either benthic or pelagic consumers (Dunn–Bonferroni pairwise comparison, *p* > 0.05 for both cases). The overall contribution of the benthic pathway did not significantly change between seasons (Mann–Whitney U Test, *p* = 0.793). When examined at the species level for those collected in both seasons within each area, there was species-specific seasonal variation in the relative importance of benthic and pelagic pathways, with spatial differences observed between ER-SR and WR (Figure 7). In spring, pelagic consumers in WR exhibited a marked increase in reliance on the benthic pathway. In contrast, the benthic reliance of consumers in ER and SR remained within a narrow range between seasons, with a slight increase observed in summer for a few benthic and benthic–pelagic species.

## 4. Discussion

This study represents a comprehensive assessment of the trophic structure of the fish food web in the SSK. Using SI tracers, we provide evidence that the seasonality of food web structures within the SSK differs between the TWC- and CDW-influenced areas. Seasonal changes in food web dynamics varied among pelagic, benthic–pelagic, and benthic consumers, with benthic consumers showing the least variability. Although our analysis included consumers from all three feeding zones (pelagic, benthic–pelagic, and benthic), most species analyzed were benthic. Isotopic niche overlap patterns indicated that during stratified conditions, reduced benthic–pelagic coupling led to a greater reliance on pelagic prey in the oligotrophic TWC waters. Additionally, we observed a notable decrease in the TP of several species during the stratified summer season. Our findings offer insights into the potential impacts of anticipated increases in water-column stratification on marine ecosystems in the TWC region.

The δ^13^C and δ^15^N values of phytoplankton in our study fell within the ranges documented for marine phytoplankton and POM in the literature [45]. The higher δ^13^C values observed in summer can be attributed to changes in species composition [45,46] and/or increased growth rates of marine phytoplankton due to longer daylight hours and abundant nitrogen and phosphorus nutrients in the shallow mixed layer above the thermocline [47]. A temperature-dependent increase in δ^13^C values of marine phytoplankton has been well-documented in temperate and subtropical seas [48,49]. The absence of such seasonal variation at SR may be related to the low saline water intrusion from the CDW during early summer. SR is closest to the CDW water mass among the three study areas. Since we collected POM only from the surface layer, its δ^13^C values reflect its source, namely, freshly produced phytoplankton, which is consistent with observations in offshore marine environments [50]. Our findings align with those reported in the ECS areas southwest of our study region [48].

The δ^15^N values of phytoplankton indicate that nitrate is the primary nitrogen source in this area [51]. Additionally, the relatively lower δ^15^N values observed during summer at ER and SR suggest significant atmospheric nitrogen fixation, as these areas are more exposed to the oligotrophic TWC than WR [51]. The consistent δ^15^N values of phytoplankton across seasons at ER and SR, combined with lower POM δ^15^N values in spring, are likely associated with two factors: increased DIN concentrations [49] and the incorporation of ^15^N-depleted POM from deeper layers due to spring mixing [52]. Despite these factors, phytoplankton δ^15^N values at ER and SR remained relatively stable across seasons. Therefore, a more plausible explanation for the lower POM δ^15^N values at ER and SR is the increased proportion of microbially degraded particles in the POM pool. In contrast, at WR, δ^15^N values for phytoplankton and POM were higher in spring than summer. This increase is likely due to enhanced nitrate utilization by phytoplankton resulting from more effective vertical mixing in the shallower WR during spring, leading to higher δ^15^N signatures. WR, being more productive than the other two oligotrophic areas [53], likely experiences a spring bloom that increases the proportion of freshly produced phytoplankton in the POM pool, thus elevating the POM δ^15^N signature of spring POM. The isotopic alignment of phytoplankton and zooplankton suggests typical source-to-consumer isotopic enrichment. However, the slightly higher ^15^N-enrichment observed at the TWC-influenced areas (SR and ER) could be related to an increased presence of nano- and pico-sized phytoplankton, which are less favored by larger copepods, potentially leading to a shift toward carnivory [54].

The SI values of fish and other consumers indicate that the trophic link between pelagic primary production and fish food webs varies with both season and location within the SSK basin. Benthic fish exhibited higher δ^13^C values compared with pelagic feeders, reflecting a greater reliance on detrital basal sources. This distinction between benthic and pelagic consumers’ δ^13^C values has been documented in marine ecosystems [55,56].

Our estimated food chain length, with a maximum TP of 4.5 for predators in the SSK, falls within the global average range for marine ecosystems (4.0 ± 0.5) [57]. The degree of benthic–pelagic coupling, as indicated by the proportion of benthic baseline in consumer tissues, supports the observed patterns of niche distributions. The consistently high contribution of benthic pathways to most fish aligns with findings in neighboring marine ecosystems [10,58]. The pronounced seasonal differences among feeding-zone groups at SR can be attributed to varying contributions of pelagic–benthic pathways across these groups. Temporal variations in trophic niches are common in mid- and high-latitude marine ecosystems [59,60], influenced by factors such as species composition, prey availability, habitat characteristics, and ontogenetic shifts. In our study, seasonal and spatial variations in isotopic niches and their overlaps offer insights into prey-consumer linkages. A larger niche often signifies a more complex food web structure with diverse trophic networks [60]. The increased niche size at ER during spring is likely due to the addition of ^15^N-depleted food resources from deeper layers, as evidenced by the broader δ^15^N distribution at ER in spring.

Our TP estimates further underscore seasonal variations in trophic positions among different consumer groups, with certain species exhibiting a notable decline in trophic position during stratified summer conditions. These shifts may be partially explained by temperature-induced changes in metabolic rates, a factor that merits further discussion. Increased water temperatures generally lead to increased metabolic rates [61], which can elevate energy demands and influence foraging behavior [17]. Consequently, species may adjust their dietary preferences, potentially shifting toward more abundant or alternative prey types available under stratified conditions, thereby altering their trophic positions [62].

A community-wide snapshot of trophic niches alone does not fully explain the causes behind the observed niche distribution. When consumers were categorized by their primary feeding zones, it became clear that pelagic and benthopelagic fish were the primary drivers of seasonal patterns in the two deeper areas (ER and SR). For example, the likelihood of finding benthopelagic and benthic fish within the pelagic niche increased by approximately 50% during summer. Conversely, the probability of encountering pelagic fish in deeper layers decreased due to reduced productivity in those areas. At WR, similar niche sizes across both seasons align with consistent niche overlap patterns among different consumer groups. In spring, with less water-column stratification, there is an increased influx of pelagic organic matter to benthic–pelagic and benthic consumers, resulting in a greater overlap between pelagic and benthic–pelagic consumer niches. In contrast, summer stratification decreases the supply of pelagic nutrients to deeper layers, reducing the reliance of benthic and benthic–pelagic fish on pelagic sources, as shown by our results on seasonal benthic–pelagic coupling.

In contrast to the oligotrophic ER and SR, the higher productivity and shallower depth at WR led to uneven impacts on different consumer groups. Pelagic consumers, such as the Japanese snapping shrimp (*A. japonica*), the gaper (*Champsodon snyderi*), and the Japanese anchovy (*E. japonicus*), which occupy lower trophic positions, exhibited greater seasonal variation compared with larger consumers at higher trophic positions. These pelagic consumers relied more on pelagic sources at WR than in the other areas, reflecting the elevated pelagic productivity at WR [53]. In contrast, benthic fish at ER and SR maintained a relatively stable reliance on benthic prey across seasons despite the deeper waters in these areas. This stability is consistent with the observation that deep continental shelf fish experience significant seasonal changes in diet due to fluctuations in pelagic resources, which exhibit larger seasonal variations compared with benthic prey [46].

Our results indicate that the fish food webs in the SSK are regulated by temperature-mediated seasonal bottom-up control, with the TWC playing a crucial role in shaping these patterns. With the anticipated increase in TWC influence due to global warming, prolonged summer stratification may lead to a more benthic-dependent food web structure in the SSK. Consequently, this shift could alter fisheries catch composition and overall production. Previous studies have shown that in the southwestern areas of our study region in the ECS, pelagic food webs at lower trophic levels are regulated by temperature-mediated bottom-up control [63,64]. However, the mechanisms driving changes in food web structure can vary over time [65]. Our study provides a snapshot of the food web dynamics in the SSK across two seasons within a year. Given the significant inter-annual variability of the SSK, food web dynamics can be highly unpredictable [53]. Therefore, continued monitoring is essential to understand how ongoing environmental changes impact the factors controlling fish food web structures in this temporally dynamic marine ecosystem.

## 5. Conclusions

This study highlights seasonal and spatial variations in trophic dynamics driven by water mass characteristics in the SKK. Our isotopic analysis reveals that benthic fish predominantly rely on detrital sources, whereas pelagic fish depend more on pelagic production. The estimated food chain length, reaching up to 4.5 trophic levels, aligns with global averages. Seasonal shifts in isotopic niches indicate changes in the contributions of pelagic and benthic pathways, with an increased pelagic influence in spring and decreased pelagic resources during summer. Spatial and seasonal differences reveal that higher productivity at WR results in more pronounced seasonal variations in pelagic consumers compared to the more stable niches of benthic consumers at ER and SR. These findings demonstrate how feeding zones and water-column stratification shape trophic niches of fish communities. Moreover, the anticipated increase in TWC influence due to global warming may lead to more benthic-dependent food webs, potentially impacting fisheries catch and production. Our comprehensive assessment underscores the importance of continued monitoring to capture the dynamic and unpredictable nature of trophic dynamics in the SSK. The isotopic mixing model proves valuable for obtaining space- and time-integrated information on assimilated prey [66] and serves as a useful tool for reconstructing trophic links among populations and food web structures [67]. While this study provides significant insights into the trophic niches of fish communities, further research involving stomach content analysis is needed to identify prey items and delineate structural variations in fish food webs under future warming scenarios. As global warming is expected to alter water-column stratification, future studies should focus on how these changes influence the balance between benthic and pelagic food webs, particularly in regions like the SSK. Investigating shifts in pelagic production, benthic reliance, and their effects on fish populations, growth rates, and food web stability will be critical for understanding and managing marine ecosystems in a changing climate.

## Figures and Tables

**Table 1:** Permutational multivariate analysis of variance (PERMANOVA) and post hoc pairwise test results of δ^13^C and δ^15^N values of nektonic consumers collected during summer and spring from three regions off the coast of the Jeju Island in the Southern Sea of Korea/northern East China Sea.

Source	df	SS	*R* ^2^	Pseudo-*F*	*p* (perm)	*p* (Adjusted)
a. δ^13^C values: Main test						
Region	2	14.616	0.087	11.178	0.000	-
Season	1	5.751	0.034	8.796	0.003	-
Feeding-zone	2	5.748	0.034	4.396	0.015	-
Region × Season	2	3.092	0.018	2.365	0.094	-
Region × Feeding zone	4	3.370	0.020	1.289	0.272	-
Seson × Feeding zone	2	0.684	0.004	0.523	0.591	-
Region × Season×Feeding zone	4	4.046	0.024	1.548	0.192	-
Residual	200	130.758	0.778	-	-	-
Total	217	168.065	1	-	-	-
b. δ^13^C values: Pairwise tests						
WR vs. ER	1	11.963	0.104	16.459	0.001	0.003
WR vs. SR	1	2.706	0.021	3.083	0.083	0.249
ER vs. SR	1	3.374	0.040	5.926	0.019	0.057
Pelagic vs. Benthopelagic	1	0.546	0.007	0.601	0.418	1.000
Pelagic vs. Benthic	1	6.585	0.053	9.790	0.001	0.003
Benthopelagic vs. Benthic	1	2.239	0.178	3.021	0.087	0.261
c. δ^15^N values: Main test						
Region	2	1.860	0.005	0.495	0.613	
Season	1	4.600	0.011	2.457	0.117	
Feeding-zone	2	2.630	0.006	0.702	0.504	
Region × Season	2	7.030	0.017	1.877	0.152	
Region × Feeding zone	4	11.780	0.029	1.572	0.178	
Seson × Feeding zone	2	0.900	0.002	0.239	0.781	
Region × Season×Feeding zone	4	6.370	0.015	0.850	0.504	
Residual	200	374.690	0.914	-	-	
Total	217	409.860	1	-	-	

## Data Availability

Most data generated or analyzed during this study are included in this published article (and its Supplementary Materials files). The dataset analyzed during the current study is available from the corresponding author on reasonable request.

## Supplementary Materials

The following supporting information can be downloaded at https://www.mdpi.com/article/10.3390/biology13121041/s1, Figure S1: Group of nektonic consumers of the Southern Sea of Korea based on the hierarchical cluster analysis of δ^13^C and δ^15^N values (‰). Color symbols represent the consumer groups based on literature. Green = Pelagic consumer; Blue = benthopelagic consumer; Black = Benthic consumers; Seasonal variation in the probabilistic niche overlap (%) among the isotopic niche three consumer groups from the Southern Sea of Korea; Table S1: The δ^13^C and δ^15^N values (‰) of selected baseline consumers for estimation of trophic position and benthic–pelagic contribution to consumer tissues; Table S2: Trophic position (TP) estimates [Median (95% credible intervals)] of different groups of consumers from the Southern Sea of Korea during summer and spring estimated by the tRopihcPositoin package in R; Table S3: Contribution [mean (95% credible intervals) of benthic prey in of different groups of consumers from the Southern Sea of Korea during summer and spring consumer tissues estimated by the tRopihcPositoin package in R.
