# Supplementary material for: Seasonal Water-Column Structure Drives the Trophic Niche of Fish Communities on a Temperate Continental Shelf
Source: Biology (Basel). 2024 Dec 12;13(12):1041. doi: 10.3390/biology13121041 (PMC11673552; doi:10.3390/biology13121041)
Supplement: Supplementary file 1 [file biology-13-01041-s001.zip › biology-3344919-supplementary.pdf]

biology

MDPI

Supplementary Materials

Seasonal Water-Column Structure Drives the Trophic Niche of Fish Communities on a Temperate Continental Shelf

Goutam Kumar Kundu [1,2], Changseong Kim [1], Jaebin Jang [1], Chung Il Lee [3], Dongyoung Kim [3], Weol-Ae Lim [4], Jung Hwa Choi [5] and Chang-Keun Kang [1,*]

[1] School of Earth Sciences and Environmental Engineering, Gwangju Institute of Science and Technology, Gwangju 61005, Republic of Korea; goutam.kundu@du.ac.bd (G.K.K.); changseong@gist.ac.kr (C.K.); wkdwoqls7@gist.ac.kr (J.J.)
[2] Department of Fisheries, University of Dhaka, Dhaka 1000, Bangladesh
[3] Department of Marine Bioscience, Gangneung-Wonju National University, Gangneung 25457, Republic of Korea; leeci@gwnu.ac.kr (C.I.L.); dongyoung@gwnu.ac.kr (D.K.)
[4] Marine Environment Research Division, National Institute of Fisheries Science, Busan 46083, Republic of Korea; limwa@korea.kr
[5] Ocean and Fisheries Development International Cooperation Institute, Pukyong National University, Busan 48513, Republic of Korea; choi2291@korea.kr
* Correspondence: ckkang@gist.ac.kr

Supplementary Information Contents

Supplementary Table S1. The $\delta^{13}C$ and $\delta^{15}N$ values (‰) of selected baseline consumers for estimation of trophic position and benthic-pelagic contribution to consumer tissues.

Supplementary Table S2. Trophic position (TP) estimates [Median (95% credible intervals)] of different groups of consumers from the Southern Sea of Korea during summer and spring estimated by the tRopihcPositoin package in R.

Supplementary Table S3. Contribution [mean (95% credible intervals) of benthic prey in of different groups of consumers from the Southern Sea of Korea during summer and spring consumer tissues estimated by the tRopihcPositoin package in R.

Supplementary Figure S1. Group of nektonic consumers of the Southern Sea of Korea based on the hierarchical cluster analysis of $\delta^{13}C$ and $\delta^{15}N$ values (‰). Color symbols represent the consumer groups based on literature. Green=Pelagic consumer; Blue=benthopelagic consumer; Black=Benthic consumers.

Supplementary Table S1. The $\delta^{13}C$ and $\delta^{15}N$ values (‰) of selected baseline consumers for estimation of trophic position and benthic-pelagic contribution to consumer tissues.

Region	Season	Pelagic baseline			Benthic baseline		
		Species	$\delta^{13}C$	$\delta^{15}N$	Species	$\delta^{13}C$*	$\delta^{15}N$*
Eastern	Spring	Copepoda *spp.*	−20.3	7.5	*Crangon hakodatei*	−18.8	5.5
Region	Summer	Copepoda *spp.*	−20.3	7.4	*Zebrias zebra*	−18.7	7.3
Southern	Spring	Copepoda *spp.*	−20.4	8.5	*Alpheus japonicus*	−18.8	6.5
Region	Summer	Copepoda *spp.*	−20.3	7.5	*Alpheus japonicus*	−18.0	6.7
Western	Spring	Copepoda *spp.*	−21.1	6.4	*Metapenaeus joyneri*	−18.2	6.7
Region	Summer	Copepoda *spp.*	−19.6	5.8	*Plesionika izumiae*	−18.6	5.7

* Adjusted with Trophic Enrichment Factor based on McCutchan et al. (2003)

Supplementary Table S2. Trophic position (TP) estimates [Median (95% credible intervals)] of different groups of consumers from the Southern Sea of Korea during summer and spring estimated by the tRopihcPositoin package in R.

Species name	Species code	Eastern Region		Southern Region		Western Region	
		Spring	Summer	Spring	Summer	Spring	Summer
**Pelagic consumer**							
Coryphaena hippurus	COH	3.5 (3.3, 3.6)					
Engraulis japonicus	ENJ			3.2 (3.0, 3.4)		3.0 (2.8, 3.2)	3.6 (3.4, 3.8)
Konosirus punctatus	KOP					4.0 (3.8, 4.1)	3.4 (3.3, 3.6)
Palaemon gravieri	PAG					3.7 (3.5, 3.9)	3.8 (3.7, 4.0)
Scomber japonicus	SCJ		3.8 (3.7, 4.0)		2.9 (2.8, 3.1)	3.9 (3.7, 4.1)	3.5 (3.3, 3.7)
Scomberomorus niphonius	SCN		3.9 (3.8, 4.1)		3.8 (3.7, 4.0)		
Sepia officinalis	SEO	3.0 (2.8, 3.1)					
Solenocera prominentis	SOP			3.4 (3.2, 3.6)		3.5 (3.3, 3.6)	3.2 (3.1, 3.4)
Thamnaconus modestus	THM	4.2 (4.0, 4.4)	3.1 (2.9, 3.3)		2.9 (2.7, 3.0)	3.3 (3.2, 3.5)	
Thunnus hynnus	THH		3.6 (3.4, 3.7)				
Trichiurus lepturus	TRL		3.6 (3.4, 3.7)	3.7 (3.5, 3.9)	3.6 (3.4, 3.7)		3.8 (3.6, 3.9)
**Benthopelagic consumer**							
Benthosema pterotum	BEP			3.2 (3.0, 3.4)		3.1 (2.9, 3.2)	
Eupymma morsei	EUM			3.0 (2.8, 3.2)			
Glossanodon semifasciatus	GLS		2.8 (2.7, 3.0)				
Harpadon nehereus	HAN					3.8 (3.6, 4.0)	
Johnius grypotus	JOG					4.1 (3.9, 4.2)	
Larimichthys polyactis	LAP			3.8 (3.6, 4.0)		3.1 (2.9, 3.3)	3.6 (3.5, 3.8)

**Species name**	**Species code**	**Eastern Region**		**Southern Region**		**Western Region**	
		Spring	Summer	Spring	Summer	Spring	Summer
Loligo beka	LOB		3.9 (3.7, 4.1)		4.2 (4.0, 4.3)		
Neobythites sivicola	NES			3.8 (3.6, 4.0)		3.5 (3.3, 3.6)	
Pampus echinogaster	PAE					4.5 (4.4, 4.7)	3.7 (3.5, 3.8)
Pennahia argentata	PEA			4.3 (4.1, 4.5)			
Psenopsis anomala	PSA		3.3 (3.2, 3.5)	3.8 (3.6, 4.0)	3.4 (3.3, 3.6)		4.3 (4.2, 4.5)
Squalus acanthias	SQA	3.5 (3.3, 3.7)					
Todarodes pacificus	TOP		3.4 (3.2, 3.5)	3.3 (3.1, 3.5)	3.5 (3.3, 3.6)	3.1 (2.9, 3.2)	
Trachurus japonicus	TRJ	3.6 (3.4, 3.8)	3.5 (3.4, 3.7)	3.4 (3.2, 3.6)	3.5 (3.4, 3.7)		4.2 (4.0, 4.3)
Zenopsis nebulosa	AEN	4.1 (3.9, 4.3)	3.5 (3.3, 3.7)				
Zeus faber	ZEF	3.7 (3.5, 3.8)	3.6 (3.4, 3.7)	4.0 (3.8, 4.2)	3.5 (3.4, 3.7)		
**Benthic consumer**							
Alpheus japonicus	ALJ			3.0 (2.8, 3.1)	3.0 (2.9, 3.2)		
Amblychaeturichthys hexanema	AMH			3.3 (3.2, 3.6)			
Amphioctopus fangsiao	AMF	3.0 (2.8, 3.2)					
Apogon lineatus	APL			3.6 (3.4, 3.8)		3.7 (3.5, 3.8)	3.5 (3.4, 3.7)
Aulopus japonicus	AUJ	2.9 (2.7, 3.0)	3.4 (3.3, 3.6)	3.5 (3.3, 3.7)			
Bembras japonica	BEJ	3.3 (3.1, 3.5)					
Caelorinchus multispinulosus	CAM			3.3 (3.1, 3.5)			
Carcinoplax longimana	CAL			3.5 (3.3, 3.7)	3.8 (3.7, 4.0)	3.4 (3.3, 3.6)	
Champsodom synderi	CHS	2.9 (2.7, 3.0)		3.0 (2.8, 3.2)			
Charybdis bimaculata	CHB					3.5 (3.4, 3.7)	3.1 (2.9, 3.2)

**Species name**	**Species code**	**Eastern Region**		**Southern Region**		**Western Region**	
		Spring	Summer	Spring	Summer	Spring	Summer
Chelidonichthys spinosus	CHSP			3.7 (3.5, 3.9)		3.5 (3.4, 3.7)	
Collichthys lucidus	COL			3.0 (2.8, 3.2)			
Collichthys niveatus	CON						3.8 (3.6, 4.0)
Conger myriaster	COM			4.1 (3.9, 4.3)		3.9 (3.7, 4.1)	4.2 (4.1, 4.4)
Crangon hakodatei	CRH	2.5 (2.3, 2.7)				3.5 (3.3, 3.6)	3.4 (3.2, 3.6)
Cynoglossus robustus	CYR						4.1 (4.0, 4.3)
Dardanus arrosa	DAA	2.8 (2.6, 3.0)		4.0 (3.8, 4.2)	3.5 (3.3, 3.6)		
Dentex tumifrons	DET	3.8 (3.6, 3.9)	3.4 (3.3, 3.6)	3.6 (3.4, 3.8)	3.6 (3.4, 3.7)		
Dipturus kwangtungensis	DIK	3.7 (3.6, 3.9)					
Doederleinia berycoides	DOB			3.2 (3.0, 3.4)		3.2 (3.1, 3.4)	
Echelus uropterus	ECU	3.9 (3.7, 4.1)					
Eopsetta grigorjewi	EOG	3.1 (2.9, 3.3)					
Erisphex pottii	EIP					2.9 (2.7, 3.0)	
Halieutaea stellata	HAS	3.4 (3.2, 3.5)					
Hoplobrotula armata	HOA			3.6 (3.4, 3.8)	3.3 (3.1, 3.4)		
Ibacus ciliatus ciliatus	IBCC				3.5 (3.3, 3.7)		
Ibacus novemdentatus	IBN	3.3 (3.1, 3.5)					
Kaiwarinus eqquula	KAE	4.1 (3.9, 4.3)	3.4 (3.2, 3.6)				
Lateolabrax japonicus	LAJ					4.5 (4.3, 4.7)	
Latreutes anoplonyx	LAA						3.0 (2.8, 3.2)
Lepidotrigla guentheri	LEG		3.1 (2.9, 3.3)				
Lepidotrigla hime	LEH	3.3 (3.2, 3.5)		3.2 (3.0, 3.4)			

**Species name**	**Species code**	**Eastern Region**		**Southern Region**		**Western Region**	
		Spring	Summer	Spring	Summer	Spring	Summer
Leptomithrax edwardsii	LEE	2.5 (2.3, 2.7)					
Lophiomus setigerus	LOS	3.9 (3.7, 4.0)		4.4 (4.2, 4.6)			
Lophius litulon	LOL	3.6 (3.4, 3.7)	3.5 (3.3, 3.6)	3.9 (3.7, 4.1)	3.8 (3.6, 3.9)	4.0 (3.9, 4.2)	4.3 (4.1, 4.4)
Macrorhmphosus scolopax	MAS	3.4 (3.3, 3.6)			3.8 (3.7, 4.0)		
Metanephrops thomsoni	MET			3.2 (3.1, 3.4)			
Metapenaeopsis barbata	MEB			3.4 (3.3, 3.6)			
Metapenaeus joyneri	MEJ					3.1 (2.9, 3.2)	
Miichthys miiuy	MIM			4.4 (4.2, 4.6)		4.1 (4.0, 4.3)	4.1 (4.0, 4.3)
Octopus minor	OCM					3.9 (3.7, 4.1)	
Octopus vulgaris	OCV	3.2 (3.0, 3.4)	2.8 (3.0, 3.1)		3.5 (3.4, 3.7)		
Oplegnathus fasciatus	OPF	3.7 (3.5, 3.8)					
Oratosquilla sp.	ORS			4.2 (4.0, 4.6)		3.6 (3.4, 3.8)	3.9 (3.7, 4.1)
Ovalipes punctatus	OVP			3.6 (3.4, 3.8)		4.1 (4.0, 4.3)	3.2 (3.0, 3.3)
Pagrus major	PAM	3.9 (3.8, 4.1)					
Pagurus ochotensis	PAO						3.5 (3.3, 3.6)
Parapenaeus fissuroides	PAF			3.0 (2.8, 3.1)			
Plesionika izumiae	PLI					3.2 (3.0, 3.4)	3.1 (2.9, 3.2)
Pleuronichthys cornutus	PLC	3.1 (2.9, 3.3)				3.4 (3.2, 3.5)	
Portunus spp	POS	2.9 (2.7, 3.0)					
Raja acutispina	RAA	3.0 (2.9, 3.2)					
Raja pulchra	RAP					3.7 (3.6, 3.9)	
Saurida microlepi	SAM	4.0 (3.9, 4.2)		3.9 (3.7, 4.1)			

**Species name**	**Species code**	**Eastern Region**		**Southern Region**		**Western Region**	
		Spring	Summer	Spring	Summer	Spring	Summer
Saurida undosquamis	SAU				3.4 (3.3, 3.6)		
Saurida wanieso	SAW	4.0 (3.9, 4.2)					
Scyliorhinus torazame	SCT	2.9 (2.8, 3.1)					
Sepia esculenta	SEE	3.4 (3.3, 3.6)	3.3 (3.1, 3.5)	3.5 (3.3, 3.6)	3.6 (3.4, 3.8)		
Sepiella japonica	SEJ	3.4 (3.2, 3.6)					
Setipinna tenuifilis	SET					3.9 (3.7, 4.1)	4.0 (3.8, 4.2)
Synodus macrops	SYM	3.5 (3.3, 3.6)	3.3 (3.1, 3.4)				
Trachysalambria curvirostris	TRC						3.8 (3.6, 3.9)
Uranoscopus japonicus	URJ			4.0 (3.8, 4.2)			
Xenocephalus elongatus	XEE			4.3 (4.1, 4.5)	3.9 (3.7, 4.0)		
Zebrias zebra	ZEZ		3.1 (2.9, 3.2)				4.2 (4.0, 4.4)
Zoarces gilli	ZOG						3.8 (3.6, 4.0)

Supplementary Table S3. Contribution [mean (95% credible intervals) of benthic prey in of different groups of consumers from the Southern Sea of Korea during summer and spring consumer tissues estimated by the tRopihcPositoin package in R.

Species name	Species code	Eastern Region		Southern Region		Western Region	
		Spring	Summer	Spring	Summer	Spring	Summer
**Pelagic consumer**							
Coryphaena hippurus	COH	0.6 (0.3, 0.8)					
Engraulis japonicus	ENJ			0.5 (0.3, 0.8)		0.8 (0.6, 1.0)	0.1 (0.0, 0.2)
Konosirus punctatus	KOP					0.0 (0.5, 0.1)	0.9 (0.6, 1.0)
Palaemon gravieri	PAG					0.4 (0.2, 0.6)	0.9 (0.7, 1.0)
Scomber japonicus	SCJ				0.2 (0.0, 0.4)	0.7 (0.5, 0.9)	0.1 (0.0, 0.3)
Scomberomorus niphonius	SCN		0.3 (0.0, 0.6)		0.6 (0.4, 0.9)		
Solenocera prominentis	SOP			1.0 (0.9, 1.0)		0.7 (0.5, 1.0)	0.1 (0.0, 0.3)
Thamnaconus modestus	THM	0.8 (0.6, 1.0)	1.0 (0.9, 1.0)		0.6 (0.3, 0.8)	0.8 (0.6, 1.0)	
Thunnus hynnus	THH		0.6 (0.3, 1.0)				
Trichiurus lepturus	TRL		0.9 (0.7, 1.0)	0.7 (0.4, 0.9)	0.2 (0.0, 0.4)		0.8 (0.5, 1.0)
**Benthopelagic consumer**							
Benthosema pterotum	BEP			0.7 (0.5, 0.9)		0.4 (0.2, 0.6)	
Eupymma morsei	EUM			0.6 (0.3, 0.8)			
Glossanodon semifasciatus	GLS		0.9 (0.7, 1.0)				
Harpadon nehereus	HAN					0.5 (0.3, 0.7)	
Johnius grypotus	JOG					0.0 (0.0, 0.1)	
Larimichthys polyactis	LAP			0.9 (0.8, 1.0)		0.5 (0.3, 0.7)	0.8 (0.4, 1.0)
Loligo beka	LOB		0.9 (0.7, 1.0)		0.9 (0.7, 1.0)		

Species name	Species code	Eastern Region		Southern Region		Western Region	
		Spring	Summer	Spring	Summer	Spring	Summer
Neobythites sivicola	NES			0.8 (0.7, 1.0)		0.6 (0.6, 0.8)	
Pampus echinogaster	PAE					0.4 (0.2, 0.6)	0.1 (0.0, 0.5)
Pennahia argentata	PEA			1.0 (0.9, 1.0)			
Psenopsis anomala	PSA		0.7 (0.4, 1.0)	0.7 (0.5, 0.9)	0.9 (0.7, 1.0)		0.7 (0.3, 1.0)
Squalus acanthias	SQA	1.0 (0.9, 1.0)					
Todarodes pacificus	TOP		0.9 (0.8, 1.0)	0.4 (0.2, 0.6)	0.7 (0.5, 1.0)	0.5 (0.3, 0.7)	
Trachurus japonicus	TRJ	0.7 (0.5, 1.0)	1.0 (0.8, 1.0)	0.8 (0.6, 1.0)	1.0 (0.9, 1.0)		0.7 (0.3, 1.0)
Zenopsis nebulosa	AEN	0.9 (0.7, 1.0)	1.0 (0.8, 1.0)				
Zeus faber	ZEF	0.7 (0.5, 0.9)	0.9 (0.8, 1.0)	0.9 (0.7, 1.0)	0.9 (0.8, 1.0)		
**Benthic consumer**							
Alpheus japonicus	ALJ			0.9 (0.8, 1.0)	0.9 (0.8, 1.0)		
Amblychaeturichthys hexanema	AMH			0.7 (0.5, 0.9)			
Amphioctopus fangsiao	AMF	1.0 (0.8, 1.0)					
Apogon lineatus	APL			0.7 (0.5, 1.0)		0.4 (0.2, 0.6)	0.3 (0.0, 0.7)
Aulopus japonicus	AUJ	0.7 (0..5, 1.0)	0.9 (0.8, 1.0)	0.7 (0.5, 1.0)			
Bembras japonica	BEJ	1.0 (0.8, 1.0)					
Caelorinchus multispinulosus	CAM			0.8 (0.6, 1.0)			
Carcinoplax longimana	CAL			0.8 (0.6, 1.0)	0.9 (0.8, 1.0)	0.7 (0.5, 0.9)	
Champsodom synderi	CHS	1.0 (0.9, 1.0)		0.9 (0.7, 1.0)			
Charybdis bimaculata	CHB					0.7 (0.5, 0.9)	0.9 (0.7, 1.0)
Chelidonichthys spinosus	CHSP			0.5 (0.3, 0.8)		0.8 (0.6, 1.0)	
Collichthys lucidus	COL			0.4 (0.2, 0.7)			

**Species name**	**Species code**	**Eastern Region**		**Southern Region**		**Western Region**	
		Spring	Summer	Spring	Summer	Spring	Summer
Collichthys niveatus	CON						0.8 (0.5, 1.0)
Conger myriaster	COM			0.8 (0.6, 1.0)		0.0 (0.0, 0.1)	0.1 (0.0, 0.4)
Crangon hakodatei	CRH	0.9 (0.7, 1.0)				0.8 (0.5, 0.9)	0.7 (0.3, 1.0)
Cynoglossus robustus	CYR						0.4 (0.0, 0.8)
Dardanus arrosa	DAA	1.0 (0.9, 1.0)		0.9 (0.7, 1.0)	0.9 (0.8, 1.0)		
Dentex tumifrons	DET	0.7 (0.4, 0.9)	0.9 (0.7, 1.0)	0.5 (0.3, 0.7)	0.3 (0.1, 0.5)		
Dipturus kwangtungensis	DIK	1.0 (0.8, 1.0)					
Doederleinia berycoides	DOB			0.6 (0.4, 0.8)		0.4 (0.2, 0.6)	
Echelus uropterus	ECU	0.7 (0.5, 0.9)					
Eopsetta grigorjewi	EOG	0.9 (0.8, 1.0)					
Erisphex pottii	EIP					0.5 (0.3, 0.7)	
Halieutaea stellata	HAS	0.9 (0.7, 1.0)					
Hoplobrotula armata	HOA			0.7 (0.5, 1.0)	0.9 (0.7, 1.0)		
Ibacus ciliatus ciliatus	IBCC				0.9 (0.8, 1.0)		
Ibacus novemdentatus	IBN	1.0 (0.9, 1.0)					
Kaiwarinus eqquula	KAE	0.9 (0.8, 1.0)	0.9 (0.7, 1.0)				
Lateolabrax japonicus	LAJ					0.3 (0.1, 0.5)	
Latreutes anoplonyx	LAA						0.1 (0.0, 0.2)
Lepidotrigla guentheri	LEG		0.9 (0.7, 1.0)				
Lepidotrigla hime	LEH	0.8 (0.6, 1.0)		0.5 (0.3, 0.7)			
Leptomithrax edwardsii	LEE	0.8 (0.5, 1.0)					
Lophiomus setigerus	LOS	0.8 (0.7, 1.0)		0.7 (0.5, 1.0)			

**Species name**	**Species code**	**Eastern Region**		**Southern Region**		**Western Region**	
		Spring	Summer	Spring	Summer	Spring	Summer
Lophius litulon	LOL	0.9 (0.8, 1.0)	0.9 (0.7, 1.0)	0.2 (0.0, 0.4)	0.8 (0.7, 1.0)	0.7 (0.5, 0.9)	0.6 (0.0, 1.0)
Macrorhmphosus scolopax	MAS	0.9 (0.7, 1.0)			0.8 (0.7, 1.0)		
Metanephrops thomsoni	MET			1.0 (0.9, 1.0)			
Metapenaeopsis barbata	MEB			1.0 (0.9, 1.0)			
Metapenaeus joyneri	MEJ					0.9 (0.8, 1.0)	
Miichthys miiuy	MIM			1.0 (0.9, 1.0)		0.5 (0.3, 0.8)	0.9 (0.8, 1.0)
Octopus minor	OCM					0.6 (0.3, 0.8)	
Octopus vulgaris	OCV	1.0 (0.8, 1.0)	0.7 (0.8, 1.0)		0.9 (0.8, 1.0)		
Oplegnathus fasciatus	OPF	0.9 (0.7, 1.0)					
Oratosquilla sp.	ORS			0.8 (0.6, 1.0)		0.3 (0.1, 0.5)	0.9 (0.8, 1.0)
Ovalipes punctatus	OVP			0.9 (0.7, 1.0)		0.7 (0.5, 0.9)	0.7 (0.2, 1.0)
Pagrus major	PAM	0.9 (0.7, 1.0)					
Pagurus ochotensis	PAO						0.4 (0.0, 0.9)
Parapenaeus fissuroides	PAF			1.0 (0.9, 1.0)			
Plesionika izumiae	PLI					0.9 (0.7, 1.0)	0.8 (0.4, 1.0)
Pleuronichthys cornutus	PLC	1.0 (0.9, 1.0)				0.6 (0.4, 0.8)	
Portunus spp	POS	0.8 (0.8, 1.0)					
Raja acutispina	RAA	0.9 (0.7, 1.0)					
Raja pulchra	RAP					0.5 (0.3, 0.7)	
Saurida microlepi	SAM	0.8 (0.6, 1.0)		0.4 (0.2, 0.6)			
Saurida undosquamis	SAU				0.6 (0.4, 0.8)		
Saurida wanieso	SAW	0.4 (0.1, 0.6)					

**Species name**	**Species code**	**Eastern Region**		**Southern Region**		**Western Region**	
		Spring	Summer	Spring	Summer	Spring	Summer
Scorpaena neglecta	SCNE	0.9 (0.8, 1.0)					
Sepia esculenta	SEE	0.9 (0.8, 1.0)	1.0 (0.9, 1.0)	0.9 (0.8, 1.0)	1.0 (0.9, 1.0)		
Sepia officinalis	SEO	0.9 (0.7, 1.0)					
Sepiella japonica	SEJ	0.9 (0.7, 1.0)					
Setipinna tenuifilis	SET					0.0 (0.0, 0.1)	0.0 (0.0, 0.2)
Synodus macrops	SYM	0.7 (0.4, 0.9)	0.9 (0.7, 1.0)				
Trachysalambria curvirostris	TRC						0.1 (0.0, 0.4)
Uranoscopus japonicus	URJ			0.9 (0.7, 1.0)			
Xenocephalus elongatus	XEE			0.8 (0.6, 1.0)	0.8 (0.7, 1.0)		
Zebrias zebra	ZEZ		0.9 (0.7, 1.0)				0.5 (0.1, 1.0)
Zoarces gilli	ZOG						0.0 (0.0, 0.2)

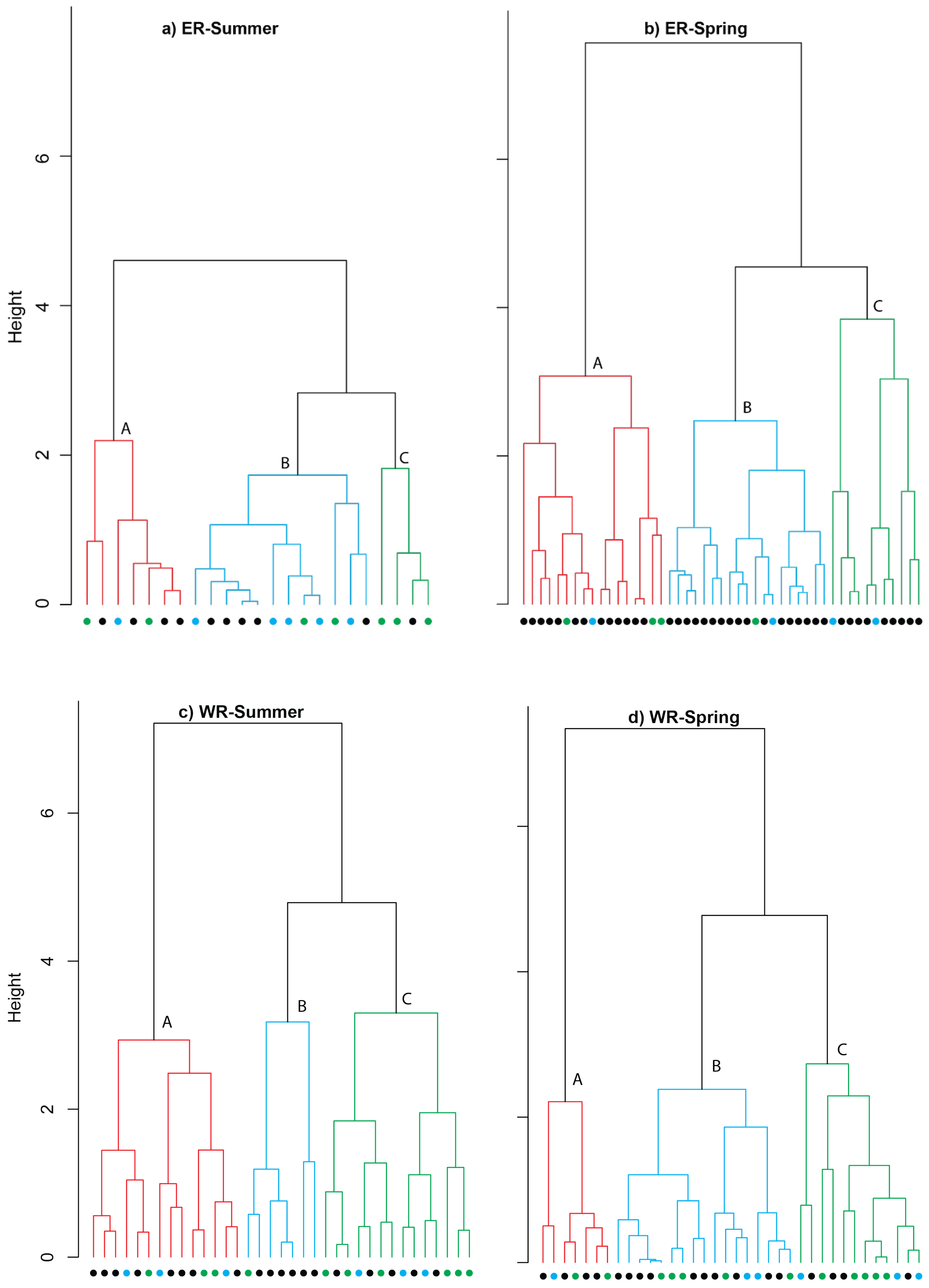
a) ER-Summer
b) ER-Spring
c) WR-Summer
d) WR-Spring
Height
0
2
4
6
A
B
C

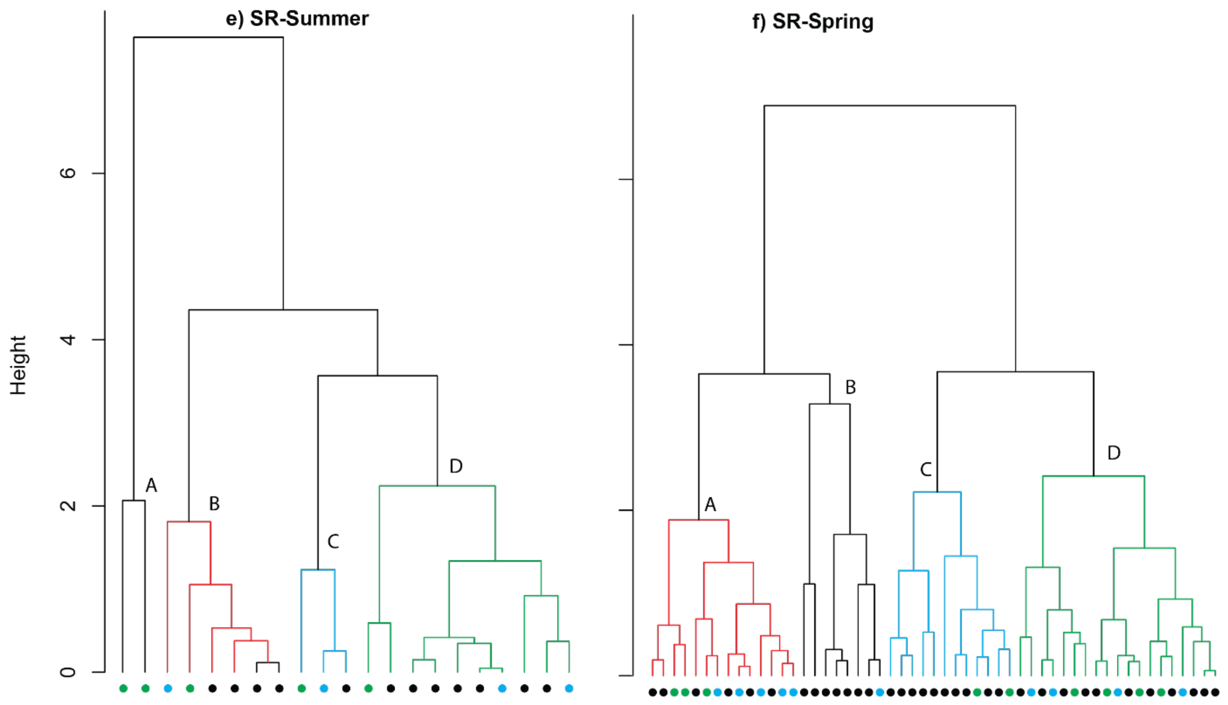

Supplementary Figure S1. Group of nektonic consumers of the Southern Sea of Korea based on the hierarchical cluster analysis of δ^{13}C and δ^{15}N values (‰). Color symbols represent the consumer groups based on literature. Green=Pelagic consumer; Blue=benthopelagic consumer; Black=Benthic consumers.
